# Optimization of the Non-Local Means Algorithm for Breast Diffusion-Weighted Magnetic Resonance Imaging Using a 3D-Printed Breast-Mimicking Phantom

**DOI:** 10.3390/life15091373

**Published:** 2025-08-29

**Authors:** Soungmo Park, Seong-Hyeon Kang, Youngjin Lee

**Affiliations:** 1 Department of Health Science, General Graduate School of Gachon University, 191, Hambakmoe-ro, Yeonsu-gu, Incheon 21936, Republic of Korea; bananafood@gachon.ac.kr; 2 Department of Radiological Science, Gachon University, 191, Hambakmoe-ro, Yeonsu-gu, Incheon 21936, Republic of Korea

**Keywords:** diffusion-weighted magnetic resonance, non-local means, breast cancer, 3D-printed breast-mimicking phantom

## Abstract

Diffusion-weighted magnetic resonance (DWMR) images were acquired using a custom-designed, 3D-printed breast-mimicking phantom. The smoothing factor of the non-local means (NLM) algorithm was then optimized for noise reduction. Phantoms were fabricated using polylactic acid, polyethylene terephthalate, and various concentrations of polyvinylpyrrolidone. DWMR images were obtained across b-values ranging from zero to 2000 s/mm^2^. Based on image contrast, the NLM algorithm was applied to the b = 1000 s/mm^2^ image, testing smoothing factors from 0.001 to 0.150. The NLM algorithm’s performance was quantitatively evaluated using a single DWMR image acquired from this custom phantom. At the optimized smoothing factor, the signal-to-noise ratio (SNR) improved from 96.87 ± 3.42 to 215.81 ± 4.18, and the contrast-to-noise ratio (CNR) from 43.63 ± 2.97 to 131.98 ± 3.56, representing 2.22-fold and 3.02-fold enhancements, respectively. No formal statistical tests were conducted as the analysis was based on a single acquisition. The optimized NLM algorithm also outperformed conventional denoising methods—median, Wiener, and total variation—in both noise suppression and contrast preservation. These findings suggest that the NLM algorithm with optimized parameters is likely to be more effective than existing approaches for enhancing breast DWMR image quality. However, further validation using in vivo patient datasets is essential to confirm its diagnostic utility and clinical generalizability because of the absence of tissue heterogeneity, motion, and physiological noise in the phantom environment.

## 1. Introduction

Breast cancer is the most common malignant tumor among women worldwide. According to global breast cancer statistics, approximately 2.26 million new cases were diagnosed in 2020. Additionally, about 685,000 women died from breast cancer, highlighting the severity of the disease. The sharp increase in the number of breast cancer patients underscores the growing demand for early detection and the development of effective treatment strategies. It also emphasizes the need to strengthen healthcare infrastructure and raise public awareness globally [1].

In the diagnosis of breast cancer, diffusion-weighted magnetic resonance (DWMR) imaging is a non-invasive technique effective in identifying necrotic regions within malignant tumors that exhibit restricted diffusion, thereby enhancing diagnostic accuracy [2]. Nevertheless, DWMR has inherent limitations, with image noise being a primary concern. Noise originates from thermal energy-induced random fluctuations of electrons during image acquisition and quantization errors during analog-to-digital conversion, causing visual discomfort, degrading image sharpness, and obscuring critical clinical features [3]. This issue becomes more pronounced at high b-values, where increased sensitivity to diffusion leads to reduced signal intensity from normal tissues [4]. Additionally, DWMR is susceptible to false-positive findings, as benign conditions such as hematomas, fibroepithelial lesions, mastitis, and abscesses may mimic restricted diffusion, and to false negatives, as certain malignancies like mucinous carcinoma may exhibit minimal diffusion restriction [5]. Lesion detectability is also influenced by lesion size, anatomical location, and acquisition parameters, resulting in some tumors being undetectable. Despite these challenges, no optimal denoising approach for breast DWMR has been established, highlighting the need for advanced post-processing techniques.

In MRI, magnitude images reconstructed from complex data exhibit Rician-distributed noise, which becomes signal-dependent and non-Gaussian in low signal-to-noise ratio (SNR) regimes, complicating post-processing [6,7]. Rician noise arises when complex-valued Gaussian noise in raw MRI data is transformed into magnitude images, resulting in a biased, signal-dependent distribution that especially affects low-intensity regions. Therefore, improving image quality by reducing noise is an important research objective, as noise—particularly at high b-values—can obscure differences in apparent diffusion coefficient (ADC) values between malignant and benign lesions, reducing the reliability of BI-RADS assessment [8]. To address this, post-processing algorithms have gained attention for improving SNR and contrast-to-noise ratio (CNR).

Classical denoising algorithms such as median, Wiener, and total variation (TV) filters have been widely applied in medical image processing. However, their applicability to DWMR—particularly in textured tissues such as the breast—is limited. Specifically, the median filter is effective in removing salt-and-pepper noise but tends to excessively smooth fine tissue structures when over-applied to MR images. Similarly, the Wiener filter, designed to suppress Gaussian noise, is not well suited for Rician-distributed noise and may blur edges and high-frequency textures. Although the TV algorithm preserves edges, it can introduce staircase artifacts in relatively smooth regions [9].

In comparison, NLM filtering better preserves structural fidelity in images with complex textures, such as fibroglandular breast tissue. Moreover, the NLM algorithm offers stable, data-independent performance and low computational cost, demonstrating practical clinical utility across diverse imaging environments [10,11]. However, the NLM algorithm also has limitations. Its denoising performance is highly sensitive to the choice of smoothing factors, and inappropriate parameter selection may lead to either residual noise or over-smoothing. In addition, NLM can be computationally demanding for large datasets when not optimized, which restricts its use in real-time clinical workflows.

These limitations have motivated the development of deep learning–based denoising techniques, which aim to overcome the parameter sensitivity and computational burden of traditional NLM methods by learning noise characteristics directly from data. Recent advances in deep learning–based denoising methods, such as NAFNet-MRI, have shown outstanding performance across various MR image modalities [12,13,14,15]. However, despite growing attention, deep learning–based denoising methods face substantial barriers to clinical adoption. Unlike the NLM algorithm, which operates without prior training data, these models require large-scale, high-quality labeled datasets that are difficult to obtain due to strict ethical and privacy regulations. Furthermore, their performance often degrades when applied to unseen scanner types, magnetic field strengths, and imaging protocols, revealing poor generalization and limited robustness in heterogeneous clinical environments [16].

Therefore, NLM remains a practical choice in clinical settings, as it offers robust, data-independent performance without the need for extensive training datasets and provides effective denoising under heterogeneous imaging conditions. However, while NLM algorithms have been applied to various MR image modalities, including dynamic contrast-enhanced (DCE) MR images, their application to breast DWMR remains limited [17]. This is because DWMR has a distinct noise profile due to both the Rician distribution and signal attenuation at increasing b-values. To address these issues, this study optimizes the NLM algorithm for breast DWMR images using a breast-mimicking phantom.

## 2. Materials and Methods

### 2.1. Phantom Production

In this study, a custom-designed phantom simulating a breast structure was fabricated to enhance the accuracy and reproducibility of the experiments. For MR image suitability, it is essential to ensure both the precision and consistency of the results and to use nonmagnetic materials. Additionally, structural deformation and distortion during the fabrication process were minimized [18].

The upper support structure of the breast-mimicking phantom was fabricated using polylactic acid (PLA) filament on an Ultimaker S5 3D printer (Ultimaker B.V., Utrecht, The Netherlands). Printing parameters included: nozzle diameter, 0.4 mm; layer height, 0.1 mm; infill density, 20%; printing temperature, 210 °C; and bed temperature, 60 °C. During MR image scanning, the phantom was maintained at room temperature, ~22 °C, to ensure signal stability and minimize thermal drift. The lower support structure contained distilled water only; no T_1_-modifying agents such as CuSO_4_ were added to minimize background signal interference and isolate the effect of tissue-mimicking materials on diffusion properties.

A critical aspect of phantoms is their ability to replicate the actual breast environment. Studies have reported that polyvinylpyrrolidone (PVP) can simulate the diffusion characteristics of various breast tissues [19,20]. PVP phantom units mimicking breast lesions, particularly at higher concentrations like 40%, have been shown to yield apparent diffusion coefficient (ADC) values consistent with restricted diffusion. For instance, reported ADC values for 40% PVP solutions, depending on temperature, are typically in the range of 557 × 10^−6^ mm^2^/s to 640 × 10^−6^ mm^2^/s.

Our study aimed to focus on ADC values characteristic of malignant breast tumors, which typically exhibit restricted diffusion. While the characterization of such phantoms is often performed at 1.5 T, and our study utilized a 3.0 T MR image system, it has been reported that there is no significant difference in ADC values for breast lesions between 1.5 T and 3.0 T [21]. Moreover, mean ADC values for malignant lesions generally fall within ranges such as 600 × 10^−6^ mm^2^/s or 1050 × 10^−6^ mm^2^/s [22,23].

To replicate this clinically relevant malignant range, four types of tissue-mimicking materials were prepared by adjusting the PVP concentrations at 10% intervals, ranging from 10% to 40%. The 40% PVP concentration was selected as the most representative for analysis due to its consistent ability to mimic the restricted diffusion observed in malignant tumors across various studies, providing the most reliable ADC value for our experimental objectives. This reflects the clinical relevance of diffusion restriction as a key malignancy indicator in breast cancer diagnosis and underscores its importance in early detection and treatment planning [24].

### 2.2. MRI Equipment and DWMR Image Acquisition

A MAGNETOM VIDA 3.0 T MRI scanner (Siemens Healthineers, Erlangen, Germany) was used to acquire high-resolution images. To optimize the SNR and ensure clinical relevance, an 18-channel dedicated breast coil was employed, following manufacturer recommendations and previously validated breast DWMR protocols [25,26]. Imaging was performed using a combination of generalized autocalibrating partially parallel acquisition (GRAPPA) and echo-planar imaging (EPI) techniques to minimize diffusion-induced artifacts, which are known to minimize diffusion-induced artifacts in breast MRI [27]. The major imaging parameters were as follows: repetition time (TR) = 4500 ms, echo time (TE) = 108 ms, slice thickness = 3.0 mm, no slice gap, number of excitations (NEX) = 2, matrix size = 256 × 256, and field of view (FOV) = 230 × 230 mm^2^ [28]. The receiver bandwidth was set at 271 Hz/pixel to reduce susceptibility artifacts while maintaining image fidelity [29]. The native in-plane resolution was approximately 0.9 × 0.9 mm^2^. Scanner-enabled interpolation yielded a displayed voxel size of 0.4 × 0.4 × 3.0 mm^3^. Although this exact voxel size is specific to our scanner, previous studies recommend in-plane resolutions around 1 mm or smaller (for example, pixel area approximately 0.4 mm^2^) for breast MRI to improve lesion morphology visualization, ADC measurement reliability, and post-processing accuracy [30]. Fat suppression was achieved using a spectral adiabatic inversion recovery (SPAIR) technique. Phase encoding was performed in the left-to-right direction to acquire coronal images with a parallel imaging acceleration factor of 4. For consistent signal acquisition, the phantom was positioned at the center of the coil during scanning. The diffusion sensitivity, b, was incrementally adjusted from 0 to 2000 s/mm^2^ at 200 s/mm^2^ intervals, and DWMR images were acquired at 0, 200, 400, 600, 800, 1000, 1200, 1400, 1600, 1800, and 2000 s/mm^2^ covering the clinically relevant ADC range for distinguishing malignant from benign tissues [31]. This broad sampling strategy provides detailed information on signal attenuation, supporting consistent denoising evaluation and reflecting clinically relevant ADC values [32]. Among the 11 acquired coronal images, those with minimal distortion caused by the EPI artifacts were selected for further analysis. In this study, no additional measures were implemented to evaluate slice-to-slice variability or scan repeatability. Instead, to minimize variability, the phantom was positioned consistently at the center of the coil during all acquisitions, and slices with minimal EPI distortion were selected for analysis.

### 2.3. NLM Algorithm

**Figure 1:**
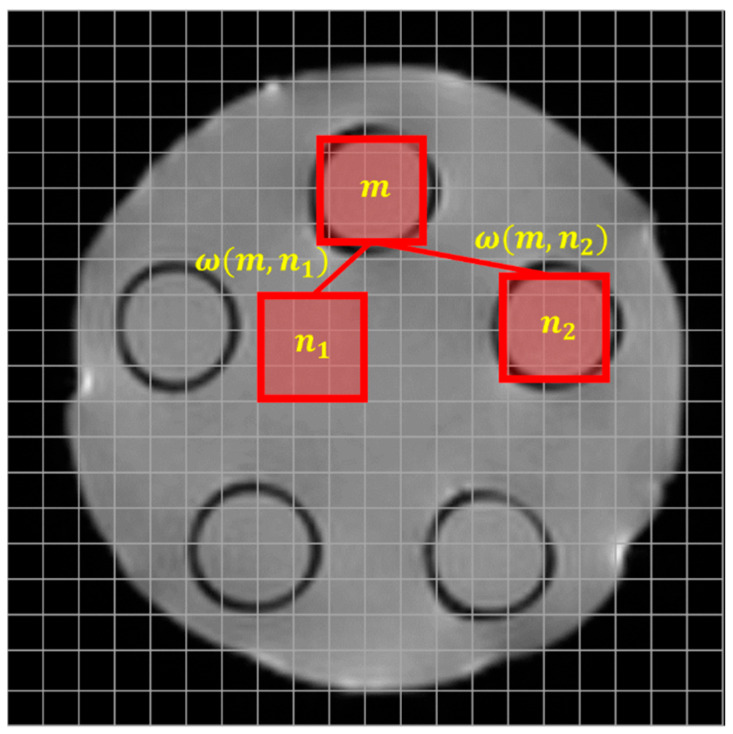
Schematic diagram illustrating the noise reduction process of the non-local means algorithm.

The NLM algorithm is a nonlinear denoising method that removes noise based on the similarity between patches, as illustrated in Figure 1. This is based on the concept that images possess inherent redundancy, wherein similar patterns and structures may recur across different regions of the image.

The basic formulation of the NLM algorithm for given locations $\left(m,n\right)$ is as follows [33]:

(1) $$NL\left[f\right]\left(m\right)=\;\sum_{n=1}\omega \left(m,n\right)f(n),$$

When calculating the pixel value at position m, the weight $\omega \left(m,n\right)$ assigned to the pixel at position n is defined as:

(2) $$\omega \left(m,n\right)=\;\frac{1}{Z(m)}e^{-\frac{{\left\|v\left(k_{m}\right)\;-\;v(k_{n})\right\|}_{2}^{2,a}}{d^{2}}},$$

In these expressions, the normalization factor $\frac{1}{Z(m)}$ ensures that the sum of all the weights equals one. This prevents the dominance of specific weights and maintains a balance among the selected reference patches, thereby avoiding abrupt changes in image contrast [34]. Additionally, the Euclidean distance ${\left\|v\left(k_{m}\right)\;-\;v(k_{n})\right\|}_{2}^{2,a}$ quantifies the similarity between a noisy patch and a reference patch, whereas parameter a determines the tolerance for such differences. By contrast, d represents the smoothing factor. A larger d increases the sensitivity to patch differences, thereby enhancing noise removal; however, excessive smoothing may lead to the loss of image details. Therefore, the appropriate selection of d according to the image characteristics is crucial [35]. In this study, various smoothing factors were applied to optimize the NLM algorithm parameters to achieve optimal performance for breast DWMR images. Studies identified the computational inefficiency of conventional NLM weight calculations and proposed an improved formulation [36]:

(3) $$\omega \left(m,n\right)=\;\frac{1}{Z(m)}\sum_{n=1}e^{-\frac{G_{a}(\lambda ){\left\|f\left(m+\lambda \right)\;-\;f(n+\lambda )\right\|}_{2}^{2}}{d^{2}}},$$

This formulation considers spatial displacement λ within patches, replacing the parameter a with $G_{a}(\lambda )$, which represents the variance of a Gaussian distribution with respect to spatial displacement. Correspondingly, the Euclidean distance term is modified to ${\left\|f\left(m+\lambda \right)\;-\;f(n+\lambda )\right\|}_{2}^{2}$. This new approach enables a more refined measurement of patch similarity, thereby achieving superior denoising performance compared with that of conventional methods. Although the NLM algorithm demonstrates excellent denoising performance, it has a major drawback in that it requires substantial computational resources owing to the need to calculate vector distances in two dimensions. To address this limitation, an improved version of the NLM algorithm was proposed. This improved NLM optimizes the computation by transforming a two-dimensional image into a one-dimensional representation, thereby significantly reducing computational complexity. This improvement reduces memory consumption and enhances the temporal resolution, thereby enabling fast image processing. The weight calculation formulas used in the improved NLM algorithm are as follows:

(4) $$\hat{\omega }\left(m,n\right)=\;\frac{1}{Z\left(m\right)}S_{i}(f\left(m+P\right)\;-\;f\left(n\;-\;P\right)),$$

(5) $$S_{i}\left(p\right)=\sum_{\tau =0}^{p}e^{-\frac{{\left\|f\left(\tau \right)-f(\tau +\tilde{\lambda })\right\|}_{2}^{2}}{d^{2}}},$$

Here, $S_{i}$ is a summation function used to measure similarity between patches, P is the patch size in one dimension, p is the window size for similarity search, τ denotes the patch location in one dimension, and $\tilde{\lambda }$ represents the displacement distance from τ to the reference patch.

The NLM algorithm was implemented in MATLAB R2022a and executed on a workstation with an Intel Core i7-11700K CPU (3.6 GHz) and 32 GB RAM, without GPU acceleration. The processing time for a single 2D slice (512 × 512) was approximately 11 s. The patch size was set to 5 × 5, based on previous optimization studies demonstrating improved denoising performance and computational efficiency with similar parameters [37].

### 2.4. Quantitative Evaluation

To quantitatively analyze the performance of the NLM algorithm, noise levels were evaluated using *SNR* and *CNR*, while high-frequency signal restoration was assessed by measuring signal intensity. Here, the signal refers to the average intensity of the MRI signal induced by the tissue and is defined as the mean pixel value within the designated ROI. By contrast, noise refers to irregular fluctuations within an image and is defined as the standard deviation of the pixel values within the same ROI. Based on these definitions, previous studies have derived *SNR* and *CNR* as follows [38,39]:

(6) $$SNR=\;\frac{Region\;signal\;intensity}{Background\;noise\;standard\;deviation},$$

(7) $$CNR=\frac{Region\;signal\;intensity-background\;signal\;intensity}{\sqrt{{Region\;standard\;deviation}^{2}+{background\;standard\;deviation}^{2}}},$$

Quantitative image analysis was performed to evaluate changes in *SNR* and *CNR* across varying b-values, with all image processing conducted in MATLAB. Images were selected from the 40% PVP solution region based on minimal distortion and clear tissue contrast. Given the study’s focus on optimizing and comparing *SNR* and *CNR* after NLM application to breast DWMR images, regions of interest (ROIs) were manually placed by a single observer in both the 40% PVP area and the adjacent homogeneous background. Each ROI was uniformly defined as 25 × 25 pixels, and the mean and standard deviation were calculated accordingly. Given that the application of the NLM algorithm inevitably leads to some loss of resolution, this study also evaluated the tradeoff between resolution and noise suppression by jointly analyzing the *CNR* and signal intensity profiles. *CNR* serves as a quantitative indicator of the degree of contrast between the ROI and background, even when signal intensity distributions become more uniform because of smoothing; a higher *CNR* indicates better-maintained contrast. Moreover, the signal intensity profile visualizes pixel value changes at the boundaries between different tissues, serving as an effective measure of image resolution. High-resolution images exhibit steep transitions at the tissue boundaries, whereas low-resolution images show gradual curves [40,41]. For this analysis, the ROI was set to include the boundary between the 40% PVP solution and the surrounding background.

To evaluate the performance of the optimized NLM algorithm, it was compared with representative conventional noise reduction methods. The median, Wiener, and TV algorithms were selected as comparators. Each algorithm was applied under identical conditions, and the DWMR images of the same slice used for the NLM evaluation were employed to maintain consistency. For the median and Wiener algorithms, the kernel size was set to 5 × 5, considering the balance between detail preservation and noise suppression. For the TV algorithm, the regularization parameter was set to 0.2 to preserve image boundaries. All algorithms were applied under identical preprocessing and postprocessing conditions. The performance of each algorithm was quantitatively assessed using *SNR* and *CNR* values. This comparison demonstrates the superior *SNR* and *CNR* performance of the NLM algorithm, supporting its potential applicability in breast DWMR imaging.

All reported *SNR* and *CNR* values were derived from a single acquisition, with standard deviations reflecting pixel-wise variability within the defined ROIs, not inter-scan variability. Inter-observer variability was not assessed, and no repeated measurements were performed as only a single phantom model was used. Consequently, formal statistical testing was not applied to compare means, and *SNR*/*CNR* values are presented as single calculated metrics rather than distributions with standard deviations, which limits the generalizability of the results to some extent.

## 3. Results

### 3.1. Quantitative Evaluation Results of DWMR Images

First, the ADC values of the 40% PVP region were calculated by varying the b-values of the acquired DWMR images. The resulting ADC values ranged from 991 × 10^−6^ mm^2^/s to 1024 × 10^−6^ mm^2^/s, which fall within the range previously reported for malignant tumors. Based on these results, all subsequent algorithm implementations and performance evaluations were conducted using a 40% PVP concentration as the reference. In the acquired DWMR images, an overall decrease in both signal intensity and noise was observed as the b-value increased. The *SNR* initially increased, reaching a maximum of 123.30 at b = 600 s/mm^2^, and subsequently decreased at higher b-values. This trend suggests that b = 600 s/mm^2^ provides an optimal balance between signal and noise. The *CNR* exhibited a similar pattern, peaking at 43.63 at b = 1000 s/mm^2^. This indicates the point at which the contrast between the ROI and background was most pronounced, corresponding to the greatest difference in pixel intensity between the two regions. Because contrast differentiation between lesions and normal tissues is critical in DWMR imaging, the algorithm applied in this study was based on b = 1000 s/mm^2^. Under these conditions, the *SNR* and *CNR* were measured as 96.87 and 43.63, respectively [Table 1].

### 3.2. Quantitative Evaluation Results of NLM-Optimized Images

**Figure 2:**
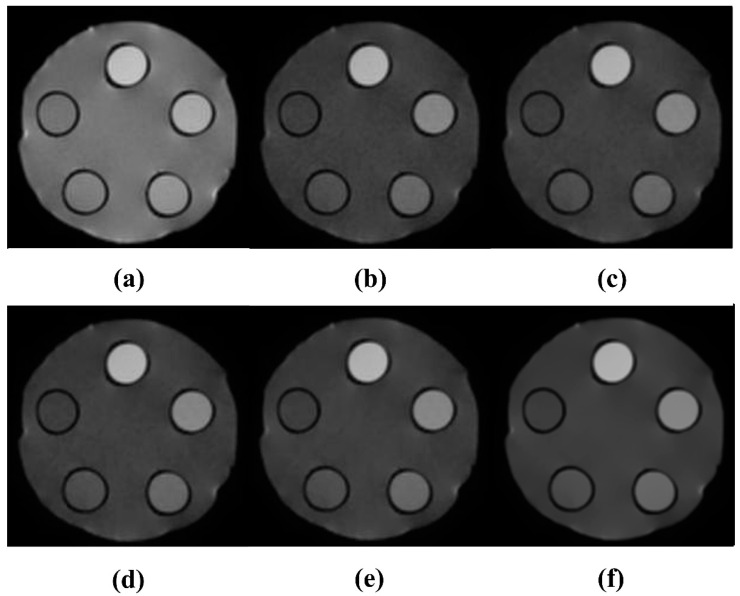
Optimization of the smoothing factor in b = 1000 s/mm^2^ diffusion-weighted magnetic resonance images: (**a**) 0.001, (**b**) 0.013, (**c**) 0.040, (**d**) 0.080, (**e**) 0.120, (**f**) 0.150.

**Figure 3:**
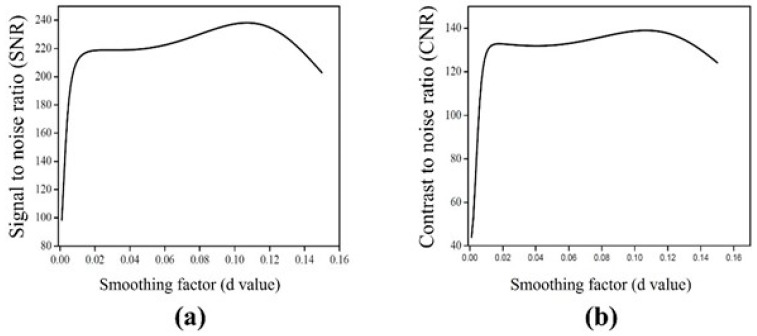
Changes in *SNR* and *CNR* with varying smoothing factors in diffusion-weighted magnetic resonance images: (**a**) Signal to noise ratio (SNR) and (**b**) contrast to noise ratio (CNR).

To evaluate the noise reduction effect of the NLM algorithm, it was applied to a DWMR image acquired at b = 1000 s/mm^2^, where the highest *CNR* was observed while varying the smoothing factor. During implementation, a 5 × 5 pixel patch, centered on each noise-affected pixel and extending two pixels in all directions, was defined. The search window for similar-patch identification was set to 15 × 15 pixels, covering seven pixels in all directions. The smoothing factor ranged from 0.001 to 0.150 in intervals of 0.001. Each setting was evaluated by calculating the *SNR* and *CNR*. As shown in Figure 2, increasing the smoothing factor led to enhanced noise suppression. In particular, both *SNR* and *CNR* exhibited a steep increase until the smoothing factor reached 0.013. In the range between 0.012 and 0.13, only minor fluctuations were observed, with overall performance remaining relatively stable. However, when the smoothing factor exceeded 0.13, both *SNR* and *CNR* showed a gradual decline. This trend can be visually confirmed in Figure 3. At a smoothing factor of 0.013, noise was effectively suppressed while contrast was clearly enhanced. In contrast, when higher smoothing factors were applied, noise was excessively removed, and excessive smoothing of boundary signals led to distortion of adjacent tissue signals.

**Figure 4:**
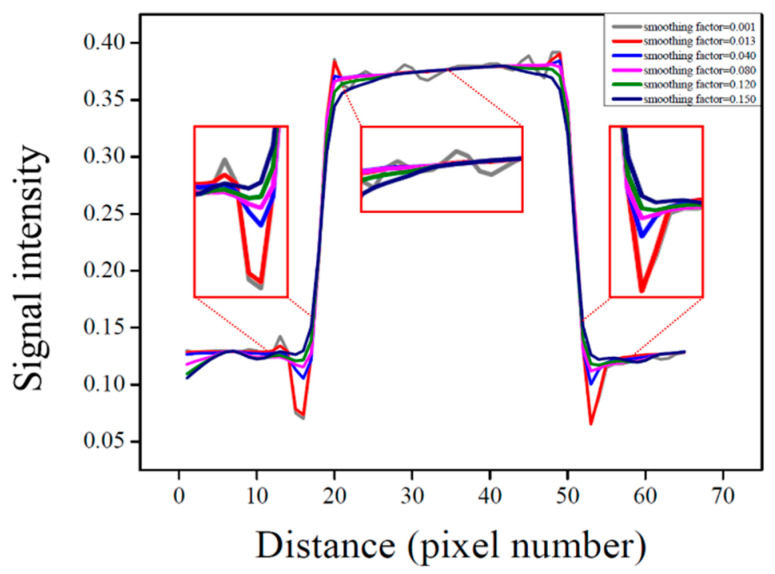
Signal intensity profile of pixel values as a function of the smoothing factor, with magnified views at tissue regions and boundaries.

Similarly, the signal intensity profile analysis shown in Figure 4 confirms the smoothing effect as the smoothing factor increases, indicating effective noise suppression. Furthermore, it was confirmed that when the smoothing factor was set to a value below 0.13, edge signals experienced less distortion due to smoothing, allowing for the preservation of high-frequency signals.

However, within single-tissue regions, pixel value variation showed minimal change after a smoothing factor of 0.013, and at tissue boundaries, blurring became noticeable when the smoothing factor exceeded 0.040. This finding suggests that application of the algorithm leads to resolution loss. Considering the balance between noise reduction and resolution preservation, a smoothing factor of 0.013 was identified as optimal. Under this condition, the *SNR* and *CNR* were measured as 215.81 and 131.98, respectively, representing increases of 2.22-fold and 3.02-fold, respectively, compared with the pre-algorithm images. As the smoothing factor varied, the *SNR* ranged from 98.52 to 238.03, and the *CNR* ranged from 44.02 to 138.91. These results demonstrate improvements of up to 2.25-fold in *SNR* and 3.04-fold in *CNR*, compared with the original DWMR image values of 96.87 and 43.63, respectively [Table 2].

### 3.3. Comparison with Conventional Noise Reduction Algorithms

**Figure 5:**
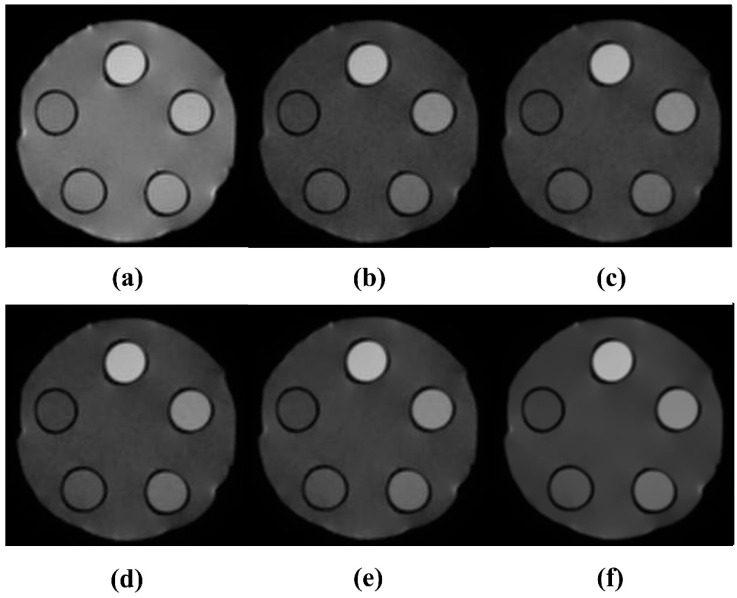
Comparison of diffusion-weighted magnetic resonance images using no algorithm, conventional algorithms, and the optimized non-local means algorithm: (**a**) b = 600 s/mm^2^, no algorithm; (**b**) b = 1000 s/mm^2^, no algorithm; (**c**) median; (**d**) Wiener; (**e**) total variation; (**f**) optimized non-local means.

Before evaluating the performance of the optimized NLM algorithm, a quantitative comparison analysis was conducted by applying the median, Wiener, and TV algorithms to the DWMR images, as shown in Figure 5.

The resulting *SNR* values for each algorithm were 150.36 for median, 185.38 for Wiener, and 138.14 for TV, whereas the corresponding *CNR* values were 57.67, 76.84, and 53.53, respectively. All algorithm-applied images demonstrated noise reduction compared with the original image, with overall improvements in both *SNR* and *CNR*. Based on these results, a direct comparison with the optimized NLM algorithm was performed. The images processed with the optimized NLM algorithm exhibited relative improvements in *SNR* of 1.43-fold, 1.16-fold, and 1.56-fold compared with the median, Wiener, and TV algorithms, respectively. Similarly, the *CNR* was enhanced by 2.28-fold, 1.71-fold, and 2.46-fold, respectively [Table 3].

**Figure 6:**
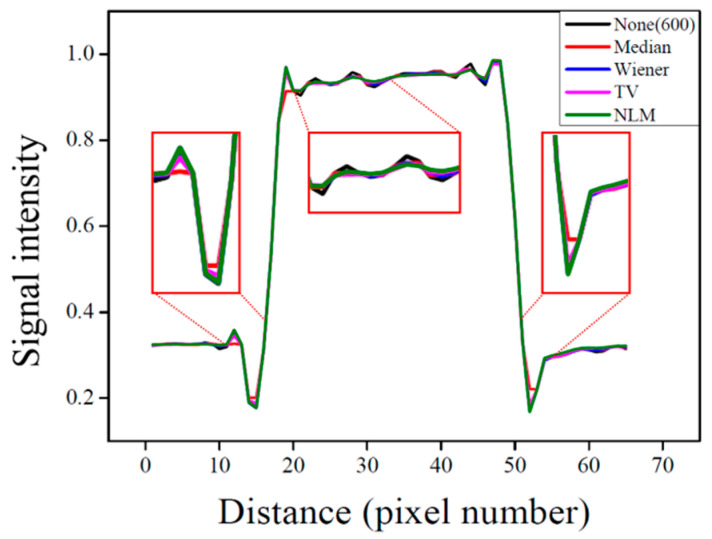
Signal intensity profiles of the optimized non-local means (NLM), the original image, and images processed with conventional algorithms, with magnified views at tissue regions and boundaries. TV, total variation.

These findings suggest that the optimized NLM algorithm achieves more effective noise reduction than the conventional methods. In addition, the degree of resolution preservation for each algorithm was analyzed by comparing the signal intensity profiles. As shown in Figure 6, all algorithm-applied images exhibited smoothing of the signal transition between the 40% PVP region and normal tissue, indicating effective noise suppression. However, for the median algorithm, a distinct signal loss was observed at the boundary region, suggesting a comparatively greater reduction in spatial resolution. In contrast, the intensity profile near the boundaries in the NLM-filtered images was found to be very similar to that of the images before filtering. This demonstrates that the NLM algorithm can preserve edge signals while selectively removing noise during the smoothing process.

## 4. Discussion

The recent increase in the number of breast cancer cases has further emphasized the importance of accurate and noninvasive diagnostic techniques. In particular, MRI is widely used in breast cancer diagnosis because of its high specificity and accuracy. Among MRI techniques, DWMR is widely regarded as an essential sequence for visualizing restricted diffusion because it can depict diffusion restriction caused by breast cancer [42,43]. However, DWMR images inherently suffer from excessive noise because of signal attenuation during acquisition, and various noise-reduction algorithms have been developed to address this limitation. Nevertheless, these conventional algorithms often exhibit insufficient noise reduction performance or cause blurring of image boundaries, thereby presenting significant challenges.

To overcome these issues, the NLM algorithm—a nonlinear approach that leverages patch-based similarity—has been proposed and reported to outperform traditional filtering methods [44]. However, optimization studies that specifically target breast DWMR images using NLM are limited. Therefore, the present study aimed to optimize noise reduction performance by applying the NLM algorithm while varying the smoothing factor and evaluating its effects on *SNR*, *CNR*, and spatial resolution.

A custom-designed phantom was used to ensure experimental precision and reproducibility. Nonmagnetic materials, such as PLA filaments and polyethylene terephthalate, were used to minimize magnetic field interference. To simulate pathological tissue environments, PVP solutions with concentrations ranging from 10% to 40% in 10% increments were used to create various diffusion conditions. For quantitative evaluation prior to algorithm application, the apparent diffusion coefficient (ADC) value of the 40% PVP region, which is expected to exhibit the most restricted diffusion characteristics, was selected as a reference. The phantom was filled with distilled water to prevent signal loss, and DWMR images were acquired under varying b-values ranging from 0 to 2000 s/mm^2^ in increments of 200 s/mm^2^.

The analysis showed that the ADC value of the 40% PVP region was within the range reported for malignant tumors in previous studies, validating its suitability as the standard condition for subsequent evaluations and algorithm applications. Image evaluations based on b-value changes revealed that both *SNR* and *CNR* increased up to a certain point before subsequently declining. The *SNR* reached its maximum value of 123.30 at a b-value of 600 s/mm^2^, whereas the *CNR* peaked at 43.63 at a b-value of 1000 s/mm^2^. These findings are attributed to the restricted diffusion characteristics of the 40% PVP solution. Although signal attenuation occurs with increasing b-values, a portion of the proton signal is preserved by successive diffusion gradients, leading to more uniform pixel intensity within the ROI and reduced noise. However, beyond a critical b-value, noise approaches the noise floor and does not attenuate further, whereas signal attenuation becomes more pronounced, resulting in decreased *SNR* and *CNR*. Therefore, a b-value of 1000 s/mm^2^, where the *CNR* was highest, was selected for subsequent algorithm applications.

**Figure 7:**
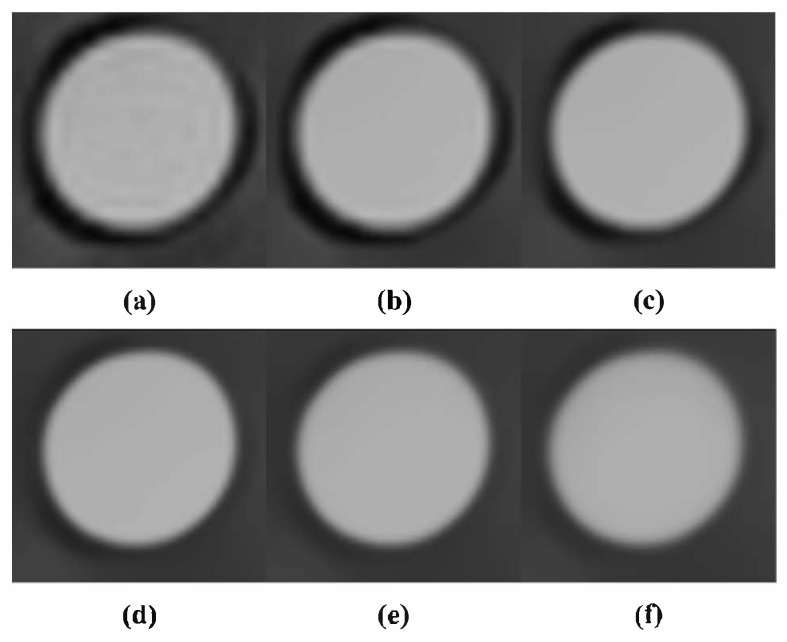
Image variation with smoothing factor at 40% PVP (**a**) 0.001, (**b**) 0.013, (**c**) 0.040, (**d**) 0.080, (**e**) 0.120, (**f**) 0.150.

Because algorithm application inherently poses a risk of resolution loss, this study also evaluated spatial resolution using both *CNR* and signal intensity profiles. *CNR* serves as a quantitative indicator reflecting both noise reduction and contrast enhancement, whereas signal intensity profiles visually depict pixel transitions at tissue boundaries, allowing for resolution assessment. The *CNR* increased sharply up to a smoothing factor of 0.013 and then stabilized, suggesting that a clear distinction between the background and ROI could still be maintained at this point. Similarly, signal intensity profile analysis showed that at a smoothing factor of 0.013, the profile exhibited large amplitude changes and distinct boundaries, confirming that this condition represents the optimal balance between noise reduction and resolution preservation. Under the optimized conditions, the *SNR* and *CNR* were measured as 215.81 and 131.98, respectively, representing 2.22-fold and 3.02-fold improvements, respectively, compared with the original images. Figure 7 presents magnified DWMR images of the phantom obtained under varying smoothing factor settings. It demonstrates that, for smoothing factors above 0.013, the contrast between the two tissue-mimicking regions progressively diminishes, accompanied by an increased blurring effect.

To evaluate the performance of the optimized NLM algorithm, conventional noise reduction algorithms (median, Wiener, and TV) were applied to images of the same coronal slice, and the results were compared. In comparing performance, all competing filters (median, Wiener, and TV) were individually optimized for kernel size and smoothing parameters based on their best achievable *SNR* and *CNR* performance, ensuring a fair comparison against the optimized NLM algorithm. Compared with the original images, the *SNR* values achieved improvements of 1.55-fold, 1.91-fold, and 1.42-fold for the median, Wiener, and TV algorithms, respectively, whereas the *CNR* values improved by 1.32-fold, 1.76-fold, and 1.22-fold, respectively. These findings suggest that the conventional algorithms offer a certain level of noise reduction. However, compared with the optimized NLM algorithm, the conventional algorithms exhibited limited overall improvements in noise reduction and *CNR* enhancement.

Considering that clear differentiation between normal tissue and lesions is critical in DWMR imaging, the superior *CNR* performance achieved by the NLM algorithm may have potential clinical relevance [45]. These results may be attributed to the inherent noise characteristics of MR images and the nature of each algorithm. MR image noise follows a Rician distribution, making the median algorithm, which is effective for salt-and-pepper noise, and the Wiener algorithm, which targets Gaussian noise, unsuitable for MR images. In addition, the effectiveness of the TV algorithm is limited when the field of view (FOV) is small, the background area is extensive, and noise removal becomes less effective.

TV algorithms perform better when distinct structures or sharp edges are present in an FOV. However, the relatively small size of the breast imposes constraints on FOV settings during imaging to balance *SNR* and resolution. Similarly, in the phantom model used in this study, limitations in FOV selection existed during image acquisition, making the TV algorithm less suitable than the NLM approach. Collectively, these findings suggest that applying the NLM algorithm with a smoothing factor of 0.013 is more effective than using conventional algorithms for noise reduction in breast DWMR.

Previous studies have demonstrated that NLM-based denoising is effective in improving *SNR* in general DWMR applications [46]. In the present study, the optimized NLM algorithm applied to breast DWMR images increased *SNR* from 97.17 to 215.81, representing an approximate 2.22-fold improvement. This gain may be particularly beneficial in breast imaging, where complex fibroglandular textures and reduced *SNR* at high b-values pose significant challenges, suggesting the optimized NLM approach has the potential to maintain effective denoising performance even in anatomically complex regions like the breast.

While deep learning–based denoising algorithms offer fast inference speeds, they typically require substantial training time, large annotated datasets, and often exhibit limited generalizability across domains or imaging protocols [47,48]. In contrast, the NLM algorithm used in this study processed a 3D volume in approximately 12 s on a standard central processing unit (CPU) (Intel i7, 16 GB RAM), without requiring retraining for new datasets and maintaining consistent performance across varying imaging conditions. This highlights NLM’s practical advantages like vendor independence and ease of deployment without the need for retraining, even if it is less powerful in terms of learning capability compared to recent deep learning–based denoisers such as MPRAGE-UNet and REBAKE-MRI. Recent studies have shown that adaptive NLM-based approaches can achieve competitive denoising performance while preserving structural details in both CT and MR images [49].

The observed improvements in *SNR* and *CNR* following algorithm application may have potential clinical implications, particularly concerning lesion detectability. A recent study on prostate DWMR reported that *SNR* enhancement via post-processing not only improved qualitative image scores but also led to changes in clinical decision-making [50]. Furthermore, reduced *SNR* at high b-values is a critical limitation for reliable ADC estimation. A phantom-based study defined an *SNR* threshold below which ADC measurements become unstable or physically invalid [51]. In the present study, all post-processed images maintained *SNR* values well above this threshold, supporting the possibility that optimized NLM filtering contributes to enhanced visual clarity.

Moreover, the ADC is highly sensitive to signal fluctuations as it is derived from signal attenuation across multiple b-values. At high b-values, noise-induced signal loss can introduce substantial bias in ADC estimation, leading to inaccurate quantification. As demonstrated by a recent deep learning-based denoising study, reducing noise can mitigate ADC bias and improve lesion conspicuity in phantom settings with known ground-truth ADC values [52]. Similarly, our optimized NLM algorithm reduced stochastic signal variability across b-values, thereby potentially improving the stability and reliability of ADC measurements. These enhanced *SNR* and *CNR* may have a potential impact on clinical interpretation by boosting lesion conspicuity and improving the fidelity of ADC measurements. This is particularly crucial in breast DWMR, where lesion boundaries often appear subtle at high b-values. Enhanced noise suppression can aid in distinguishing malignant tissue from surrounding parenchyma, thus potentially supporting lesion grading [53].

For broad clinical deployment, the NLM-based denoising framework, despite demonstrating improvements in *SNR* and *CNR* within the phantom environment, requires further validation and infrastructure development. Specifically, it should be implemented as a vendor-neutral plugin compatible with Picture Archiving and Communication Systems (PACS) platforms to ensure operability across various imaging modalities. Moreover, multi-center clinical trials involving diverse hardware configurations, imaging protocols, and patient populations are essential to evaluate the generalizability of the results and bridge the gap between controlled phantom evaluation and practical clinical application. Future work will explore the integration of the NLM-based preprocessing framework into deep learning pipelines as an initial step toward stabilizing noise levels and improving training consistency across heterogeneous MRI datasets, leveraging the complementary strengths of both NLM and deep learning approaches.

While this study offers valuable insights into noise reduction in breast DWMR imaging, it is subject to several limitations that should be acknowledged. First, our phantom model and experimental setup, while offering a reproducible and controlled environment for denoising performance evaluation, do not fully replicate the complexities of the in vivo breast environment. Specifically, the static nature of the phantom fails to account for patient-specific factors such as physiological motion artifacts, B_0_/B_1_ field inhomogeneity, or gradient nonlinearity. Moreover, the phantom’s homogeneous composition does not reflect the heterogeneous anatomical and compositional features of real breast tissue, such as fibroglandular textures or varying tissue compartments. To address this limitation, future work will explore the use of dynamic or multi-material phantoms that better emulate physiological conditions and tissue complexity. Furthermore, four PVP concentrations (10%, 20%, 30%, and 40%) were prepared to simulate a wide range of breast tissue diffusion characteristics. Among these, the 40% PVP concentration was selected for optimization because it most consistently mimicked the restricted diffusion observed in malignant tumors. Nevertheless, focusing primarily on 40% PVP may still limit generalizability, as lower concentrations (10–30%) could represent other lesion types or tumor stages with distinct diffusion properties [54,55]. While phantom validation is a necessary and valid precursor for clinical investigations, supporting the accuracy and repeatability of ADC measurements [56], the optimized NLM algorithm’s performance may differ in real clinical settings due to these additional sources of image degradation. In addition, this study utilized a 3.0T Siemens scanner; the NLM algorithm’s vendor-agnostic implementation and parameter flexibility suggest potential adaptability to other scanner platforms (e.g., GE, Philips) and field strengths (e.g., 1.5 T). However, specific tuning of smoothing parameters may be necessary to accommodate differences in noise characteristics and acquisition protocols. Although this study was carefully designed to minimize variability in phantom positioning and slice selection, the reliance on a single-slice, single-observer analysis without repeated acquisitions inherently limits the robustness and generalizability of the results. Additional slice-level experiments or simulations were not feasible within the present study due to acquisition time constraints and the phantom setup. Future work will address these limitations through multi-slice repeated measurements and simulation-based evaluations. Future research should include a wider range of phantom conditions and dynamic factors to improve clinical translatability, along with analyses of ADC values across broader PVP concentrations and optimization of NLM parameters accordingly. In addition, studies will explore the extension of the optimized NLM algorithm to other breast MRI sequences, including T2-weighted and dynamic contrast-enhanced (DCE) imaging, to evaluate its broader clinical utility.

Second, our quantitative analysis relied primarily on ROI-based metrics (*SNR* and *CNR*), which may not fully capture overall image quality. These metrics are crucial for directly assessing noise reduction and contrast enhancement within specific lesions relevant to clinical ADC calculations. However, we did not incorporate global image quality metrics such as Peak Signal-to-Noise Ratio (PSNR) or Structural Similarity Index Measure (SSIM), primarily because a true ‘ground truth’ noise-free reference image, essential for accurate PSNR/SSIM computation, is unobtainable in real-world DWMR acquisitions. Similarly, a formal quantitative assessment of edge sharpness was not performed, nor was a formal perceptual evaluation by independent observers included. While efforts were made to mitigate potential partial volume effects by careful ROI placement in homogeneous regions, these effects were not explicitly quantified or discussed. Future studies should address these limitations by incorporating broader image quality assessments, rigorous quantification of boundary preservation, and comprehensive observer studies, while also explicitly considering and mitigating partial volume effects. Moreover, this study compared the optimized NLM algorithm only with conventional filters without including any deep learning–based approaches. Although the focus was to optimize and validate the NLM algorithm, the lack of comparison with at least one state-of-the-art deep learning method may limit the strength of the conclusions regarding its relative performance. Future work should incorporate representative deep learning methods to provide a more comprehensive and balanced evaluation.

Third, the generalizability of our findings is constrained by the specific imaging conditions of this study. This research was conducted using only a 3.0 T MRI scanner from a single vendor and was limited to DWMR sequences. It remains unclear whether the optimized NLM algorithm can be generalized to scanners with different magnetic field strengths (e.g., 1.5 T), from other vendors, or to other essential breast MRI sequences (e.g., T2 fat-saturated imaging, DCE MR). Different noise characteristics, hardware performance, and sequence parameters across various systems and sequences may affect the algorithm’s effectiveness [57]. Therefore, further in vivo adaptation and validation are required to assess the robustness of the method under realistic clinical conditions, including validation across multiple vendors and field strengths.

Finally, the optimal performance of the NLM algorithm can be influenced by varying acquisition parameters. The key parameters affecting *SNR* and *CNR* can also impact the algorithm’s effectiveness [58]. Future studies should explore a broader range of acquisition settings and conduct optimization experiments under clinical conditions to ensure the practical applicability of the algorithm.

## 5. Conclusions

In this study, the optimized NLM algorithm demonstrated superior noise suppression and edge preservation in breast DWMR images compared to conventional methods. These phantom-based results suggest that the optimized NLM algorithm can enhance image quality and potentially improve lesion conspicuity in breast DWMR. However, the single-slice, single-acquisition design and absence of in vivo validation limit the robustness of the findings. Future work will include repeated phantom experiments, in vivo patient validation, and comparative evaluation with deep learning–based denoising methods to address these limitations and ensure clinical applicability.

## Figures and Tables

**Table 1:** Comparative analysis of *SNR* and *CNR* across different b-values in breast diffusion-weighted magnetic resonance images. *SNR*, signal-to-noise ratio; *CNR*, contrast-to-noise ratio.

b-Value	Signal Intensity	Standard Deviation	*SNR*	*CNR*
0	925.93	10.04	92.26	0.85
200	759.45	7.73	98.27	18.14
400	617.34	5.23	117.98	33.02
600	501.89	4.07	123.30	38.60
800	409.45	3.76	108.85	43.37
1000	332.54	3.43	96.87	43.63
1200	271.42	2.95	92.02	37.78
1400	221.70	2.87	77.36	35.14
1600	181.40	3.01	60.26	30.33
1800	148.13	3.00	49.31	25.76
2000	120.16	2.69	44.70	25.96

**Table 2:** Optimization of the smoothing factor in diffusion-weighted magnetic resonance images and evaluation based on signal intensity, standard deviation, *SNR*, and *CNR*. *SNR*, signal-to-noise ratio; *CNR*, contrast-to-noise ratio.

Smoothing Factor	Signal Intensity	Standard Deviation	*SNR*	*CNR*
0.010	332.54	3.38	98.52	44.02
0.011	332.57	1.57	211.52	128.57
0.012	332.56	1.56	213.38	130.25
0.013	332.55	1.55	214.77	131.32
0.014	332.55	1.54	215.81	131.98
0.015	332.55	1.54	216.60	132.39
0.02	332.56	1.53	217.20	132.63
0.03	332.59	1.52	218.63	132.77
0.04	332.61	1.52	218.92	132.15
0.05	332.61	1.52	219.07	131.87
0.06	332.59	1.51	220.15	132.15
0.07	332.55	1.50	222.47	133.00
0.08	332.50	1.47	225.92	134.34
0.09	332.43	1.44	230.11	135.96
0.10	332.34	1.42	234.31	137.56
0.11	332.25	1.40	237.41	138.71
0.12	332.15	1.40	238.03	138.91
0.13	332.04	1.41	234.90	137.66
0.14	331.94	1.46	227.47	134.68
0.15	331.83	1.53	216.28	130.06

**Table 3:** Comparison of *SNR* and *CNR* among images without denoising, with conventional algorithms, and with the optimized NLM algorithm. *SNR*, signal-to-noise ratio; *CNR*, contrast-to-noise ratio; TV, total variation; NLM. Non-local means.

Algorithm	*SNR*	*CNR*
None (600)	123.30	38.60
None (1000)	96.87	43.63
median	150.36	57.67
Wiener	185.38	76.84
TV	138.14	53.53
NLM (1000)	215.81	131.98

## Data Availability

Data will be made available on request.
